# A HD-Zip I transcription factor from physic nut, *JcHDZ21*, confers sensitive to salinity in transgenic Arabidopsis

**DOI:** 10.3389/fpls.2023.1097265

**Published:** 2023-02-15

**Authors:** Yuehui Tang, Jingrui Peng, Jin Lin, Miaomiao Zhang, Yun Tian, Yaqian Shang, Shuying Chen, Xinxin Bao, Qiyuan Wang

**Affiliations:** ^1^College of Life Science and Agronomy, Zhoukou Normal University, Henan, Zhoukou, China; ^2^School of Journalism and Communication, Zhoukou Normal University, Henan, Zhoukou, China

**Keywords:** HD-Zip gene family, salt stress, *JcHDZ21*, physic nut, arabidopsis

## Abstract

HD-Zip is a plant-specific transcription factor that plays an important regulatory role in plant growth and stress response. However, there have been few reports on the functions of members of the physic nut HD-Zip gene family. In this study, we cloned a HD-Zip I family gene from physic nut by RT-PCR, and named *JcHDZ21*. Expression pattern analysis showed that *JcHDZ21* gene had the highest expression in physic nut seeds, and salt stress inhibited the expression of *JcHDZ21* gene. Subcellular localization and transcriptional activity analysis showed that JcHDZ21 protein is localized in the nucleus and has transcriptional activation activity. Salt stress results indicated that *JcHDZ21* transgenic plants were smaller and had more severe leaf yellowing compared to those of the wild type. Physiological indicators showed that transgenic plants had higher electrical conductivity and MDA content, and lower proline and betaine content compared with wild-type plants under salt stress. In addition, the expression of abiotic stress-related genes in *JcHDZ21* transgenic plants was significantly lower than that in wild type under salt stress. Our results showed that ectopic expression of *JcHDZ21* increased the sensitivity of transgenic Arabidopsis to salt stress. This study provides a theoretical basis for the future application of *JcHDZ21* gene in the breeding of physic nut stress-tolerant varieties.

## Introduction

Salinity is one of the main abiotic stress factors that limit crop yields. Plants can initiate a series of corresponding stress tolerance mechanisms to resist environmental stress under abiotic stress conditions. During this process, almost all plants showed changes in the expression levels of various functional genes and regulatory protein genes. Among these genes, transcription factors can improve the tolerance of plants to extreme environmental stress by activating the expression of many downstream stress-resistant genes. The transcription factors related to abiotic stress discovered so far are mainly concentrated in the following gene family members, such as DREB, MYB, HD-Zip, WRKY and NAC (Dubos et al., 2010; Rushton et al., 2010; Harris et al., 2011; Javelle et al., 2011; Thirumalaikumar et al., 2018; Feng et al., 2020).

The HD-Zip family is a type of plant-specific transcription factor that contains a highly conserved homeodomain (HD) and a leucine zipper (LZ) domain (Johannesson et al., 2001). The HD-Zip protein forms a dimer through the LZ domain, and then acts as a transcription factor by combining its HD with a specific DNA sequence (Deng et al., 2002). According to the conservative and structural characteristics of the amino acid sequence, the family can be divided into 4 subfamilies, named HD-Zip I-IV (Henriksson et al., 2005). Most of the HD-Zip proteins related to plant stress response belong to HD-Zip I subfamily, and the expression of this family member is mainly affected by drought, salt, cold and osmotic pressure, and increasing the expression of these genes can change the tolerance of transgenic plants to extreme environmental stress (Harris et al., 2011). For example, overexpression of *ATHB-6* increases drought stress tolerance in transgenic maize by activating the expression of reactive oxygen species-related genes (Jiao et al., 2022), *PsnHDZ63* transgenic plants showed better phenotypic and physiological indicators under salt stress, indicating that increasing the expression of the *PsnHDZ63* gene altered the tolerance of transgenic plants to salt stress (Guo et al., 2021). Overexpression of maize *Zmhdz4* and *Zmhdz10* genes in rice increases the tolerance of transgenic plants to drought and salt stress (Zhang et al., 2012; Zhao et al., 2014). In addition to participating in abiotic stress regulation, HD-Zip family genes also play important roles in plant growth and development (Turchi et al., 2013; Preciado et al., 2022). *ABIG1* plays a role in leaf laminar growth as well as in adaxial-abaxial polarity establishment (Preciado et al., 2022). Overexpression of *ATHB13* affects cotyledon shape by inhibiting lateral expansion of epidermal cells in sugar-treated seedlings (Hanson et al., 2001). *OsHOX1* and *OsHOX28* regulate the local distribution of auxin by reducing endogenous auxin content, thereby regulating the size of the tiller angle (Hu et al., 2020). Although many HD-Zip genes have been cloned from different plants, the physiological and molecular mechanisms by which HD-Zip family genes regulate plant responses to salt stress, especially in salt-tolerant Euphorbiaceae species, need to be further studied.

Physic nut (*Jatropha curcas*), a Euphorbiaceae species, has high salinity tolerance, and rich in oils suitable for production of biodiesel, which is one of the most promising biomass energy plants (Montes and Melchinger, 2016). Although physic nut has strong salt tolerance, the molecular mechanism of how physic nut responds to salt stress tolerance is still unclear. In previous study, we notice that a HD-Zip I family transcription factor, we named *JcHDZ21*, which is strongly repressed expression by salinity stress (Tang et al., 2019). On this basis, we cloned the *JcHDZ21* gene and investigated its function in Arabidopsis. Up-regulation of *JcHDZ21* gene expression increases the sensitivity of transgenic Arabidopsis to salt stress, indicating that *JcHDZ21* acts as a negative regulator in the regulation of plant responses to salt stress. The results provide foundations for exploring roles of *JcHDZ21* gene in responses to salinity stress in physic nut.

## Material and methods

### Plant material and stress treatment

After the physic nut seeds germinated, the three-week-old physic nut seedlings with uniform growth were divided into three groups for abiotic stress, and each group contained twenty seedlings. Then, the physic nut seedlings in the first and second groups were used to irrigate 1/2 MS nutrient solution containing 150 mM NaCl for salinity stress treatment. After the stress treatment, the fourth leaves of five treatment time points (0 h, 2 h, 4 h, 12 h and 24 h) were selected for RNA extraction and gene expression detection. In addition, the roots, cortex stem, flowers, and leaves of the 3-week-old physic nut seedlings without stress treatment were taken for tissue expression analysis. The material was quickly frozen in liquid nitrogen and stored in a refrigerator at -80°C. The growth conditions of physic nut under normal growth and stress treatment were as follows: 16 h light/8 h dark, and the temperature was controlled at 28°C.

### JcHDZ21 protein characterization and sequence alignment analysis

Download the amino acid sequence of Arabidopsis proteins from TAIR database (https://www.arabidopsis.org/). Download the amino acid sequence of *Oryza sativa* L. and *Zea mays* L. proteins from NCBI database (https://www.ncbi.nlm.nih.gov/). SMART online software and NCBI were used to detect the conserved domain of JcHDZ21 protein. We selected HD-Zip I group proteins with positive regulator function in abiotic stress for multiple sequence alignment analysis. Amino acid multiple sequence alignment analysis was performed using the software DNAMAN XL.

### Subcellular localization of JcHDZ21 protein

Using physic nut leaf cDNA as a template, the full-length CDS sequence of the *JcHDZ21* gene (with the stop codon removed) was amplified by RT-PCR and verified by sequencing. Then connect the correct gene sequence to the subcellular localization vector to construct pBWA(V)HS-JcHDZ21-GLosgfp fusion expression vector. Finally, the constructed pBWA(V)HS-JcHDZ21-GLosgfp fusion expression vector and empty vector (control vector) were co-transformed into *Arabidopsis* protoplasts by PEG-mediated method. The isolation and extraction of *Arabidopsis* mesophyll protoplasts and the PEG-mediated transient transformation were carried out according to the method in the previously reported paper (Yoo et al., 2007). Add 100 μL of plasmid DNA to the 100 μL of protoplast suspension. Add 200 μL of PEG4000 solution to the protoplast suspension, store the tube on room temperature for 30 min. Finally, add 1 mL of W5 solution to the mixture, and culture in the dark at 23°C for 16 h, and then the fluorescence was observed under a laser confocal microscope (LSM800, Carl Zeiss).

### Transcriptional activity assay

The cDNA sequence of JcHDZ21 was combined with the GAL4 DNA binding domain on the pGBKT7 expression vector to construct the pGBKT7-JcHDZ21 recombinant expression vector. The recombinant expression vector was transferred into Y2HGold yeast competent cells by PEG/LiAc method, and cultured on SD/-Trp solid medium for 3-5 d. Then pick a single colony and inoculate it in SD/-Trp liquid medium. Taking 2.5 uL of pGBKT7-GAL4, pGBKT7, pGBKT7-JcHDZ21 bacterial solution dropwise on SD/-Trp, SD/-Trp/-His/X-a-gal and SD/-Trp/-His/-Ade/X-a-gal plates. Finally, verify transcriptional activity based on colony growth and whether it turns blue.

### Construction of plant expression vector and selection of transgenic plants

Using physic nut root cDNA as a template, the full-length cDNA sequence of *JcHDZ21* was amplified by RT-PCR and verified by sequencing. Subsequently, the target gene and plant expression vector were digested by *Kpn* I and *Xba* I. After electrophoresis, gel excision, and recovery of DNA fragments, the target gene was ligated to the binary transformation vector pCAMBIA1301 by T4 DNA ligase, and the target gene was driven by the CaMV 35S promoter. The vector was transformed into *Agrobacterium* strain GV3101, and then we transformed the constructed vector into *Arabidopsis thaliana* (Columbia ecotype) by Agrobacterium-mediated transformation of Arabidopsis using the floral dip method (Zhang et al., 2006). Homozygous plants of the T3 generation were selected in hygromycin-resistant medium. The transgenic Arabidopsis was then identified by GUS staining and RT-PCR techniques. Finally, the effective three transgenic lines were used for subsequent functional analysis.

### Analysis of salt tolerance of transgenic Arabidopsis

To avoid mechanical root damage of 4-day-old Arabidopsis seedlings during transferring for salinity treatment, sterile seeds were sown directly into 1/2 MS solid medium containing 100 mM NaCl in petri dishes for salinity stress. Then put them in the growing room to grow at 22 ± 2°C under a 16 h light/8 h dark photoperiod. After 25 days, observing the salt tolerance of wild-type and transgenic plants. Salt stress treatment experiments contained three biological replicates.

### Determination of physiological indicators of wild-type and transgenic plants

Seeds of wild-type and transgenic plants were spotted into 1/2 MS solid medium containing 0 and 100 mM NaCl in petri dishes. The leaves of wild-type and transgenic plants growth in control and salt stress conditions for 15 days were taken, washed twice with deionized water, and then the surface water of the leaves was blotted with filter paper. Accurately weigh 0.1 g and cut into filaments and place in a test tube containing 10 mL of deionized water. The test tube was then placed in a vacuum pump for 30 min. After standing at room temperature for 1 h, we used a HANNA 546808 (Italy) conductivity meter to measure the initial conductivity value and named R1. Immediately after heating the test tube in boiling water for 30 min, the running water was cooled to room temperature and shaken well, the conductance value R2 was measured. Calculate the relative conductivity using the following formula: R1/R2×100%. The betaine content was detected by ELISA kit, the specific operation method is shown in the kit instructions. The detection methods of proline and MDA content refer to the papers published by the previous research group (Tang et al., 2019).

### RNA isolation and qRT-PCR analysis

Seeds of wild-type and transgenic plants were spotted into 1/2 MS solid medium containing 0 and 100 mM NaCl in petri dishes. Then culture vessels containing the seeds were incubated at 22 ± 2°C under 16/8 h (light/dark) conditions. The leaves of wild-type and transgenic plants growth in control and salt-stressed conditions for 15 days were collected and used for qRT-PCR analysis. Plant RNA extraction kit (Magen, China) was used to extract the total RNA of each tissue in this study, and then the extracted RNA was synthesized into cDNA by TAKARA reverse transcription kit. TB Green^®^ Fast qPCR Mix (TAKARA, Beijing) was used for real-time fluorescence quantitative PCR detection, and the BIO-RAD Mini Opticon fluorescence quantitative analyzer was used for qRT-PCR reaction and analysis. Each reaction contains 100 ng cDNA, primers (10 μmol/L) 0.4 μL, SuperMix 10 μL, PassiveDye 0.4 μL, add ddH_2_O to 20 μL. The amplification program was initial denaturation at 94°C for 30 s, denaturation at 94°C for 5 s, annealing at 60°C for 15 s, extension at 72°C for 34 s, 40 cycles. All the above specific operating methods were carried out according to the kit instructions. Physic nut *JcActin* (GenBank Accession JQ806331) and Arabidopsis *Actin2* (GenBank Accession AT3G18780) were used as internal reference genes. Primer Premier 5 was used for the design of the gene-specific qRT-PCR primers. Primer sequences were shown in **Supplementary Table 1**. The specificity of qRT-PCR amplification was confirmed by melting curve analysis. Then using the 2^–ΔΔCt^ method to calculate the relative expression of genes in different samples based on the Ct value of each sample at a specific fluorescence threshold.

### Statistical analysis

The SPSS 25.0 software was used for data analysis. Significance analysis was performed using One-Way ANOVA for LSD testing of SPSS 25.0 data processing system (* P<0.05; ** P<0.01).

## Results

### *JcHDZ21* encodes a HD-Zip protein belonging to HD-Zip I subfamily

In order to study the biological function of *JcHDZ21* gene, we cloned the gene by RT-PCR using physic nut leaf cDNA as template. Sequence analysis showed that the full-length open reading frame of *JcHDZ21* was 969 bp, encoding 322 amino acids. SMART analysis revealed that JcHDZ21 contained a highly conserved HD domain and an LZ domain.

**Figure 1:**
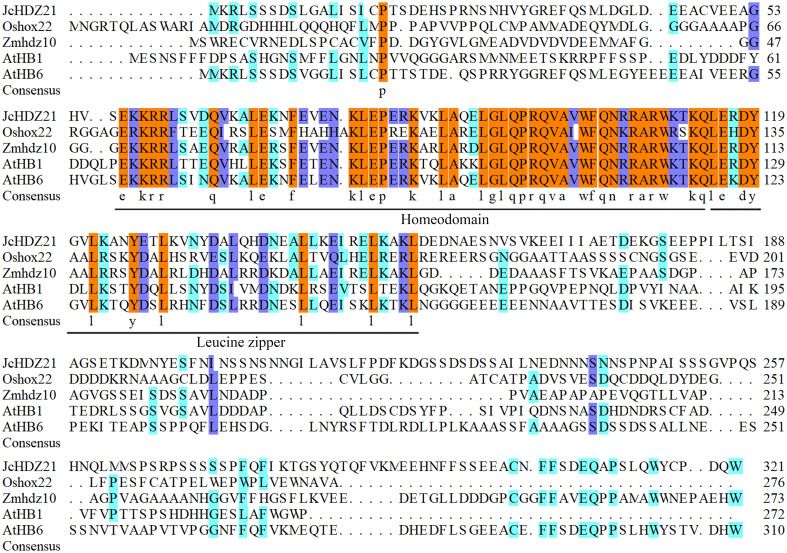
Sequence alignment analysis of JcHDZ21 and HD-Zip I family proteins that have been functionally studied. The DNAMAN XL software was used to perform the sequence alignment analysis.

We further analyzed the homology between JcHDZ21 and HD-Zip proteins of other species by DNAMAN XL software. Sequence alignment analysis showed that, like the reported HD-Zip proteins, JcHDZ21 also contained the same characteristic regions named homeodomain and leucine zipper domains (**Figure 1**). Results also showed that many amino acids residues distributed in the N-terminal and C-terminal of HD-Zip protein were variable. Taken together, these results indicated that the different functions of HD-Zip proteins in different species may be attributed to these highly variable amino acid residues.

### Expression profile of *JcHDZ21* gene

**Figure 2:**
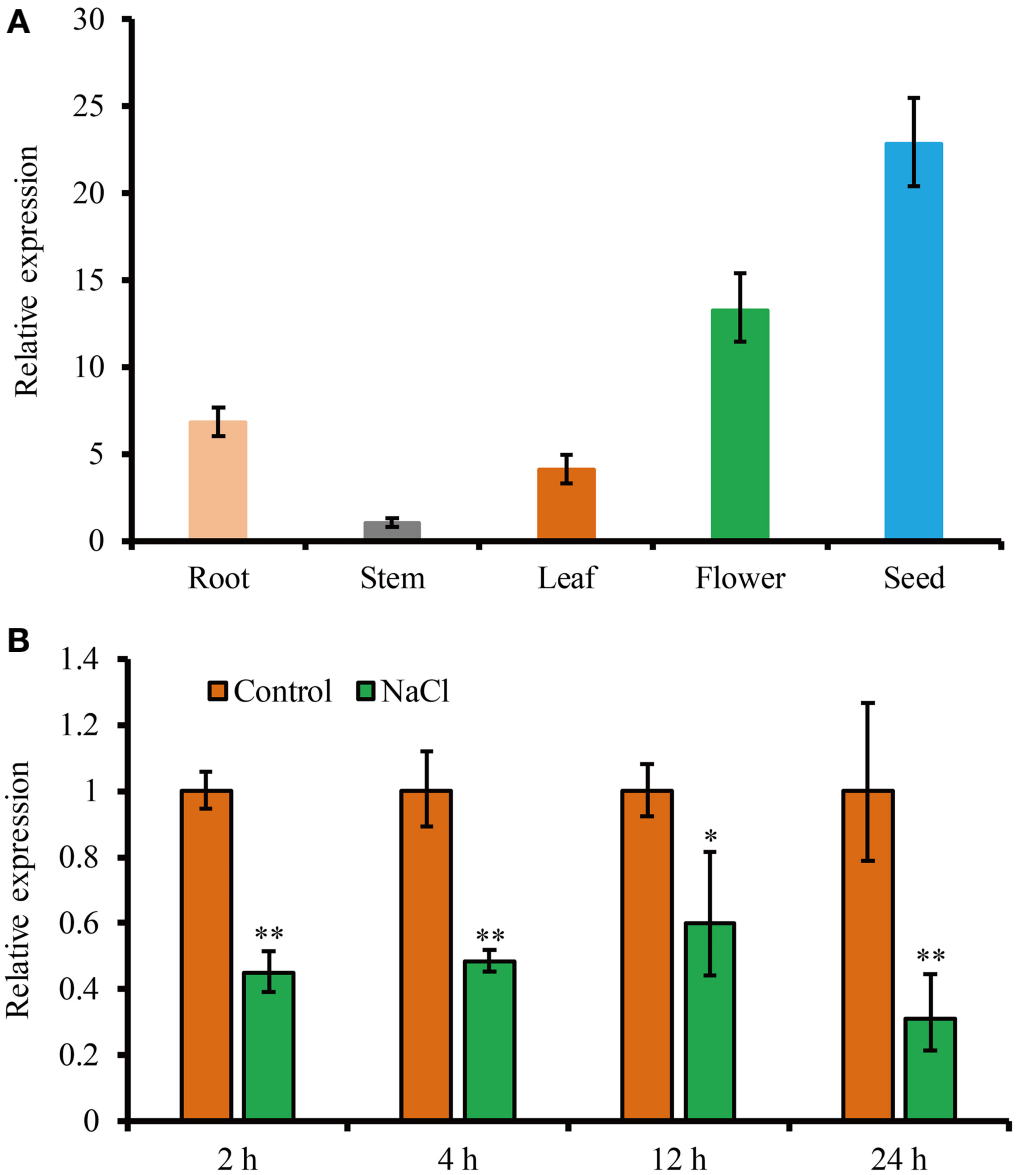
Expression level of *JcHDZ21* gene in physic nut growth in control and salt stress conditions. **(A)** Expression levels of *JcHDZ21* gene in different tissues of physic nut under normal growth conditions. **(B)** Expression level of *JcHDZ2*1 gene in physic nut leaves treated with 150 mM NaCl. Data are from three independent biological replicates, asterisks represent significant differences (p<0.01).

To study the expression profile of *JcHDZ21* gene, we analyzed the expression of *JcHDZ21* gene in roots, stems and leaves of physic nut at six-leaf stage, flowers and seeds 35 days after pollination by qRT-PCR. The results showed that the expression of *JcHDZ21* gene could be detected in all tissues of physic nut, with the highest expression in seeds, followed by flowers and roots, and the lowest expression in stems (**Figure 2A**).

In order to analyze the expression characteristics of *JcHDZ21* gene under salt stress, we further detected the expression level of *JcHDZ21* gene under salt stress. The results showed that the expression of *JcHDZ21* under salt stress was significantly lower than that in the control group (**Figure 2B**). The above results suggest that the *JcHDZ21* gene can be inhibited by salt stress, indicating that the *JcHDZ21* gene may be involved in the regulation of plants to salt stress.

### Subcellular localization of JcHDZ21 protein

**Figure 3:**
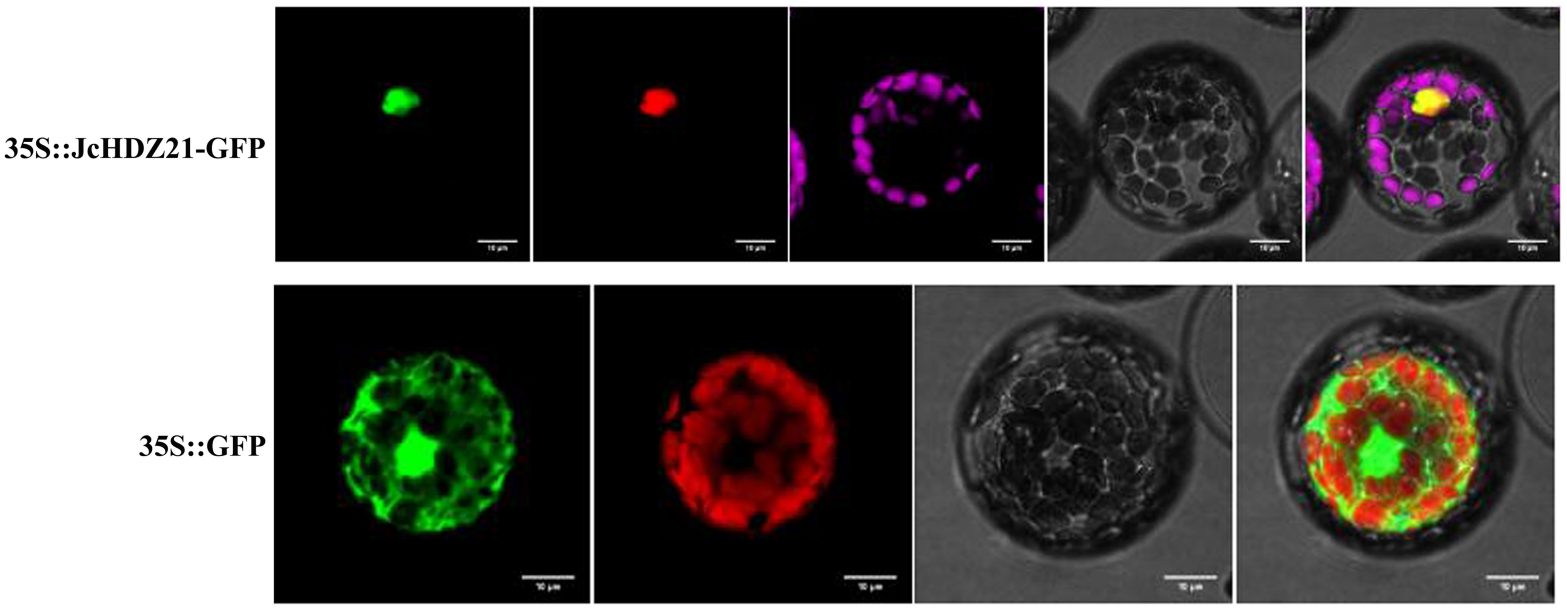
Subcellular localization of JcHDZ21 protein. 35S::JcHDZ21-GFP shows GFP, NLS-Cherry, Chlorophy II, Bright and Merge from left to right, whereas 35S::GFP shows GFP, Chlorophy II, Bright and Merge from left to right. Bars shows 10 μm.

To clarify the subcellular localization of JcHDZ21 protein, we transformed p16318hGFP-JcHDZ21 fusion expression vector and GFP empty vector plasmid into *Arabidopsis* protoplasts, and observed the subcellular localization of JcHDZ21-GFP fusion protein under laser confocal microscope. The result showed that the GFP empty control protein was expressed in all cells, while the JcHDZ21-GFP fusion protein suggested green fluorescence only in the nucleus (**Figure 3**), indicating that the JcHDZ21 protein is localized in the nucleus.

### Analysis of JcHDZ21 protein transcriptional activity

**Figure 4:**
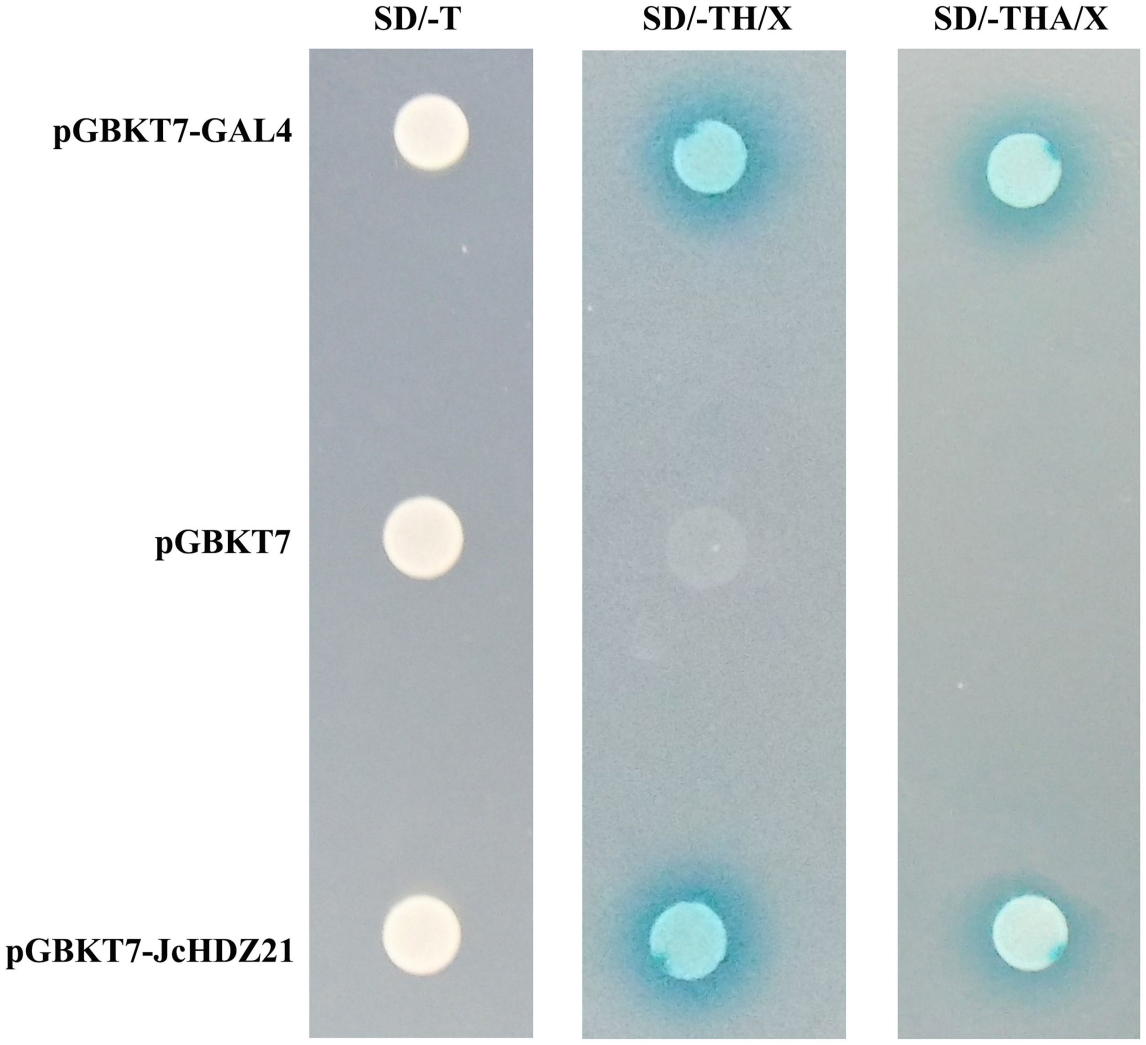
JcHDZ21 protein transcriptional activity. The left panel shows pGBKT7-GAL4, pGBKT7 and pGBKT7-JcHDZ21 grown on SD/-Trp plates for 4d, and the middle panel shows pGBKT7-GAL4, pGBKT7 and pGBKT7-JcHDZ21 grown on SD/-Trp/-His/X-a-gal plates for 4d, and the right panel shows pGBKT7-GAL4, pGBKT7 and pGBKT7-JcHDZ21 grown on SD/-Trp/-His/-Ade/X-a-gal plates for 4d.

In order to further explore the transcriptional activity of JcHDZ21 protein, we used the yeast two-hybrid system to detect the transcriptional activity of JcHDZ21 in yeast. The results showed that all the combined vectors could grow on SD/-Trp medium. On SD/-Trp/-His/X-a-gal medium, the negative control grew weakly and did not turn blue, but pGBKT7-JcHDZ21 recombinant expression vector and the pGBKT7-GAL4 plaques showed blue. However, on SD/-Trp/-His/-Ade/X-a-gal medium, the pGBKT7 vector could not grow, but the pGBKT7-JcHDZ21 recombinant expression vector and the pGBKT7-GAL4 could grow well and the plaques were blue (**Figure 4**). These results indicate that *JcHDZ21* acts as a transcriptional activator.

### Phenotypic analysis of *JcHDZ21* transgenic plants

**Figure 5:**
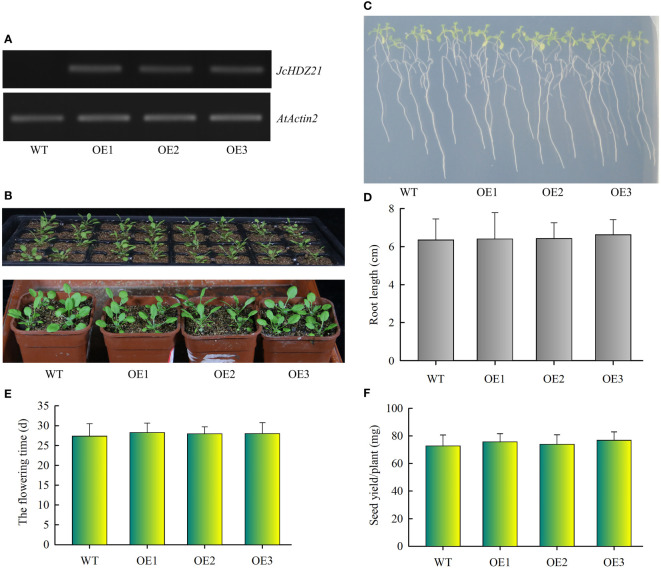
Phenotype of *JcHDZ21* transgenic Arabidopsis plants under normal growth condition. **(A)** Expression levels of *JcHDZ21* in wild-type and transgenic plants. **(B)** Growth status of wild-type and *JcHDZ21* transgenic plants at 25 days. **(C)** Root morphology of wild-type and *JcHDZ21* transgenic plants at day 10. The seeds of the sterilized wild-type and *JcHDZ21* transgenic plants were sown on 1/2 MS medium for vertical cultivation for 4 days, and the plants with consistent growth were selected and transferred to 1/2 MS medium for vertical growth for 6 days. **(D)** Root length of wild-type and *JcHDZ21* transgenic plants at day 10. Data in **(D)**: means of n = 20 ± SD from three independent experiments. **(E)** The flowering time of wild-type and JcHDZ21 transgenic plants. **(F)** Seed yield of wild-type and JcHDZ21 transgenic plants under normal growth conditions. Values represent means of n = 30 ± SD from three independent experiments.

To verify the function of the *JcHDZ21* gene, we constructed a *JcHDZ21* overexpression vector and obtained *JcHDZ21* overexpressing transgenic Arabidopsis plants. Homozygous plants of the T3 generation were selected in hygromycin-resistant medium for further study. We further detected the expression of *JcHDZ21* gene in wild-type and transgenic plants by RT-PCR. The results showed that high expression levels of *JcHDZ21* were detected in transgenic plants, but no expression was detected in wild-type plants (**Figure 5A**). We selected 4-week wild-type and transgenic plants for phenotype observation. Phenotypic analysis showed that there was no significant difference in the growth of *JcHDZ21* transgenic plants and wild-type plants (**Figures 5B, C**). Statistical data showed that *JcHDZ21* transgenic plants had no significant difference in root length compared with wild-type plants (**Figure 5D**). We further analyzed the flowering time and yield per plant of *JcHDZ21* transgenic plants. The results showed that the flowering time and yield per plant of *JcHDZ21* transgenic plants were not significantly different from those of wild-type plants (**Figures 5E, F**), indicating that ectopic expression of *JcHDZ21* gene did not affect the growth and development of transgenic Arabidopsis.

### *JcHDZ21* negatively regulates the tolerance of transgenic Arabidopsis to salt stress

**Figure 6:**
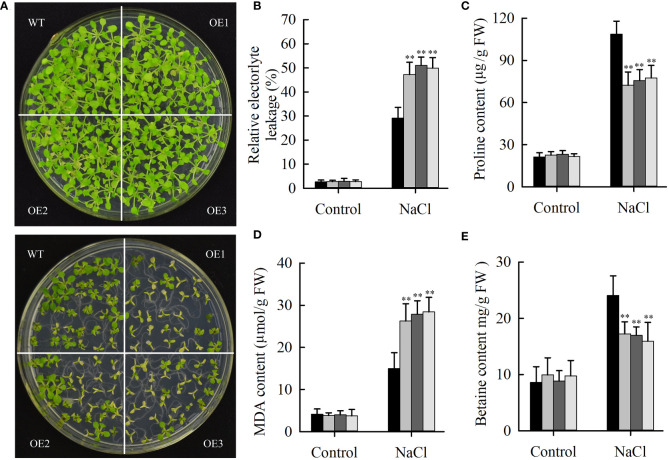
Phenotypic and physiological indicators analysis of wild-type and *JcHDZ21* transgenic plants growth in control and salt stress conditions. **(A)** Phenotypes of wild-type and *JcHDZ21* transgenic plants at 25 days growth in control and salt stress conditions. **(B)** Relative electrolyte leakage of leaves of wild-type and overexpressing plants after stress treatment and under non-stress conditions. **(C)** Proline content of leaves of wild-type and overexpressing plants after stress treatment and under non-stress conditions. **(D)** MDA content of leaves of wild-type and overexpressing plants after stress treatment and under non-stress conditions. **(E)** Betaine content of leaves of wild-type and overexpressing plants after stress treatment and under non-stress conditions. Data in **(B–E)**: means of n = 21 ± SD from three independent experiments, asterisks above the bars indicate significant differences from wild-type controls at p < 0.01according to Duncan’s multiple range test.

Salt stress can repress the expression of *JcHDZ21* gene, indicating that *JcHDZ21* might be involved in plant response to salt stress. To prove this hypothesis, we directly spread the sterilized transgenic and wild type Arabidopsis seeds onto 1/2 MS medium containing 100 mM NaCl for 25 days. The results showed that the leaves of transgenic and wild-type Arabidopsis showed different degrees of yellow and smaller symptoms, but the degree of yellow of wild-type plants leaves was significantly lower than that of transgenic Arabidopsis plants, and the leaves of transgenic Arabidopsis were smaller than that of wild-type plants (**Figure 6A**). The growth status and survival rate of transgenic Arabidopsis plants were significantly lower than those of wild-type plants. The survival rates of *JcHDZ21* transgenic plants were 22.58%, 21.24% and 19.27%, respectively, while the survival rate of wild-type plants reached 76.21%. The above results indicate that the heterologous expression of physic nut *JcHDZ21* gene significantly enhances the sensitivity of transgenic Arabidopsis to salt stress.

Some physiological indicators were also tested in transgenic and wild-type plants under normal growth and salt stress conditions. The results showed that salinity stress increased MDA, proline and betaine content, and relative electrolyte leakage, whereas wild-type plants had higher proline and betaine content, lower relative electrolyte leakage and MDA content compared with transgenic plants under salinity stress (**Figures 6B–E**). In contrast, no significant differences in these stress response indicators between wild-type and transgenic plants were observed under non-stress conditions. The above results show that overexpression of *JcHDZ21* increases the sensitivity of transgenic Arabidopsis to salt stress, which may be partly caused by changing these physiological indicators. Taken together, these results strongly suggest that *JcHDZ21* acts as a negative regulator in responses to salt stress.

### *JcHDZ21* alters the expression of abiotic stress genes under salt stress

**Figure 7:**
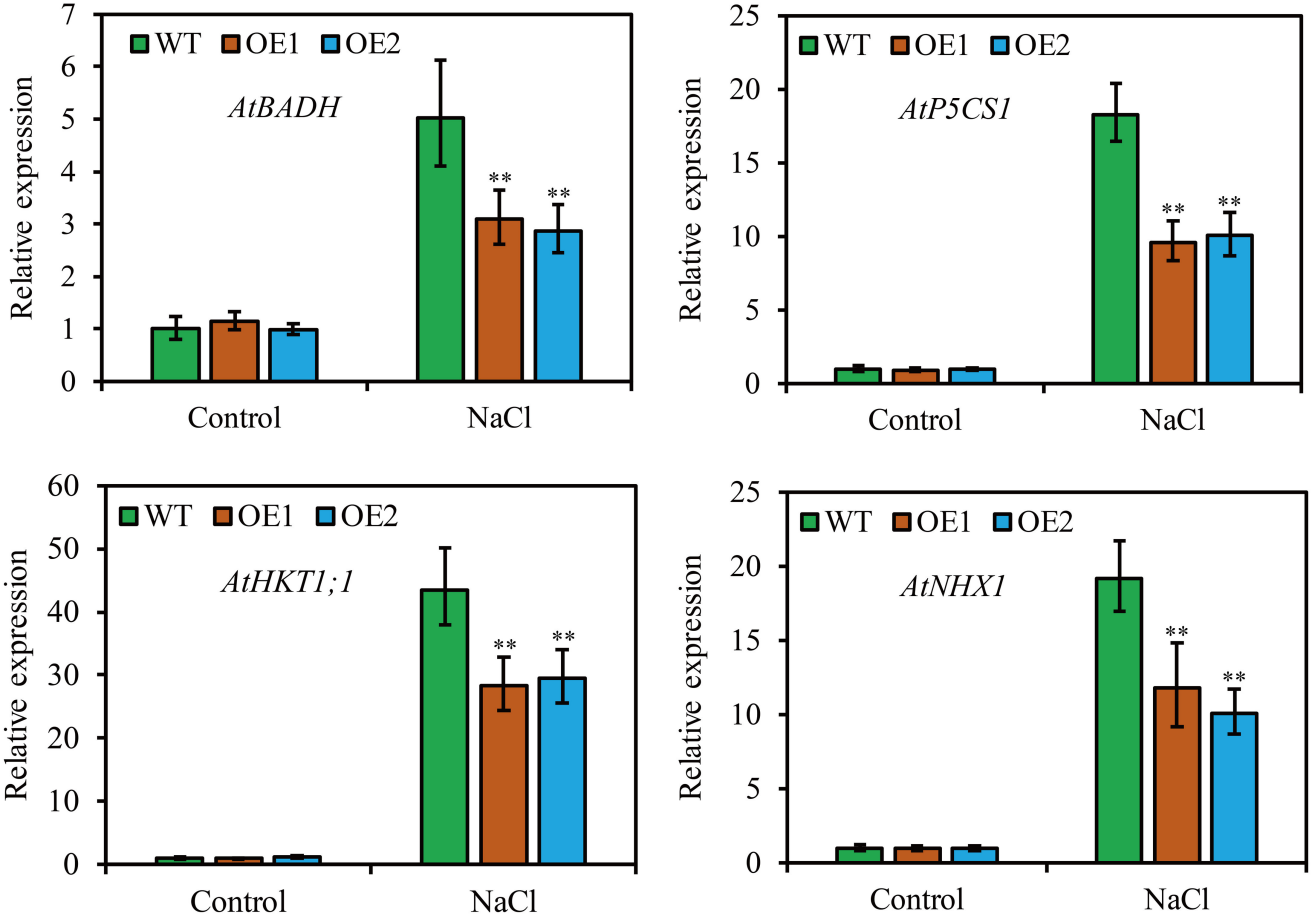
Expression levels of abiotic stress-related genes in wild-type and overexpressing plants under salt stress and non-stress conditions. Each experiment contains three biological replicates, and each with two technical replicates (means of n = 6 ± SD, asterisks above the bars indicate significant differences from wild-type controls at p < 0.01).

To clarify the molecular mechanism of *JcHDZ21* regulating Arabidopsis in response to salt stress, we further detected the expression levels of abiotic stress-related genes by qRT-PCR method, such as *AtP5CS1*, *AtNHX1*, *AtBADH* and *AtHKT1;1*. The results showed that plants growth in control condition, these abiotic stress-related genes had no significant differences among different plants. However, the expression of these abiotic stress-related genes was significantly downregulated in *JcHDZ21* transgenic plants compared to wild type when plants were exposed to salt stress (**Figure 7**).

## Discussion

HD-Zip group I transcription factors, as the main type in the HD-Zip transcription factor family, can respond to abiotic stresses by participating in various biological processes (Gong et al., 2019; Zhao et al.,2021). The properties of HD-Zip transcription factors closely related to abiotic stress have been widely reported in the model plant Arabidopsis and other plants (Perotti et al., 2021). For example, ectopic expression of the Arabidopsis *AtHB6* gene increases drought stress tolerance in transgenic maize (Jiao et al., 2022); the expression of the maize HD-Zip transcription factor gene *Zmhdz10* and the cotton transcription factor gene *MdHB7-like* enhanced the salt tolerance of the transgenic plants (Zhao et al., 2014; Zhao et al., 2021). After the physic nut genome sequencing was completed, a total of 32 HD-Zip family genes were identified in physic nut (Wu et al., 2015), but little is known about the regulatory function of HD-Zip transcription factors in physic nut. To date, only a few genes related to HD-Zip transcription factors have been identified in physic nut, such as *JcHDZ16* (Tang et al., 2019), which both alter the ability of transgenic plants to tolerate abiotic stress. Therefore, we cloned a salt stress-responsive gene *JcHDZ21* in physic nut and analyzed the function of this gene in Arabidopsis. In future research, we will use CRISPR/Cas9 genome editing technology to construct *jchdz21* mutant physic nut plants to obtain more salt-tolerant physic nut species. Thus, our results may provide foundations to further promote application of *JcHDZ21* gene in physic nut breeding.

When plants are under salt stress, some substances that regulate osmotic pressure (such as proline and betaine) will be induced to synthesize to adapt to the stress. The accumulation of proline and betaine in plants under salt stress is an important physiological phenomenon that is beneficial to the growth of plants under stress (Ganjavi et al., 2021; Hosseinifard et al., 2022). Their contents are positively correlated with plant salt tolerance and can protect plant cell membrane systems from abiotic stress (Ganjavi et al., 2021; Hosseinifard et al., 2022). Maize transcription factor *ZmWRKY114* enhances the sensitivity of transgenic Arabidopsis to salt stress by regulating proline accumulation (Bo et al., 2020). *GmNAC06* alters the salt tolerance of transgenic soybean by promoting the accumulation of proline and betaine (Li et al., 2021). Similarly, our study found that increasing the expression of the *JcHDZ21* gene increased the sensitivity of transgenic Arabidopsis to salt stress, and the accumulation of proline and betaine in *JcHDZ21* transgenic plants induced by salt stress was significantly lower than that in wild-type plants (**Figure 6**), indicating that proline and betaine may be the factors that confer lower salt stress tolerance in *JcHDZ21* transgenic plants. Increases in malondialdehyde (MDA) and relative electrical conductivity caused by abiotic stress are also important indicators of plant oxidative damage (Xie et al., 2019). In our study, compared with wild type, the relative conductivity and MDA content of *JcHDZ21* transgenic plants induced by salt stress were significantly increased (**Figures 6B–E**). Therefore, we speculate that the increase in relative conductivity and MDA content induced by salt stress may be one of the reasons for the weaker salt stress tolerance of *JcHDZ21* transgenic plants. JcHDZ21 protein has transcriptional activation activity, suggesting that JcHDZ21 may interact with other transcriptional repressors to regulate the process of plant response to salt stress.

In addition to physiological indicators that can reflect the parameters of plants responding to adversity stress, some abiotic stress genes also play an important role in plant responses to abiotic stresses when plants encounter adversity stress. *AGL16* negatively regulates salt stress progression by altering the expression of abiotic stress-related genes (Zhao et al., 2021). *Oshox22* alters the tolerance of transgenic plants to salt stress by promoting the expression of abiotic stress-related genes (Zhang et al., 2012). Similarly, our study found that the expression of abiotic stress-related genes (*AtP5CS1*, *AtNHX1*, *AtBADH* and *AtHKT1;1*) in *JcHDZ21* transgenic plants was significantly lower than that in wild type under salt stress conditions (**Figure 7**). *P5CS1* has been identified as the major contributor to stress-induced proline accumulation (Feng et al., 2016), and ectopic expression of the Arabidopsis *P5CS1* gene increases salt stress tolerance in transgenic tobacco by regulating proline accumulation (Ibragimova et al., 2015). *AtHKT1;1* gene plays an important role in regulating the process of plant response to salt stress, and increasing the expression of this gene increases the tolerance of transgenic plants to salt stress (Plett et al., 2010). Increased expression of the *AtNHX1* gene alters the tolerance of transgenic plants to salt stress (Asif et al., 2011). *AtBADH* increases salt stress tolerance in transgenic plants by altering betaine content (Zhou et al., 2008). Taken together, *JcHDZ21* may affect the biosynthesis of proline and betaine by regulating the expression of proline and betaine metabolism genes under salt stress, thereby increasing the sensitivity of transgenic plants to salt stress. Another possibility is that *JcHDZ21* may alter the tolerance of transgenic plants to salt stress by regulating the expression of abiotic stress-related genes.

## Conclusion

*JcHDZ21* is a member of the HD-Zip I family, which is highly expressed in seeds and responds to salt stress. JcHDZ21 protein is localized to the nucleus and has transcriptional activity. Under the condition of salt stress, the damage degree of salt stress on *JcHDZ21* transgenic Arabidopsis was significantly higher than that of wild type. This study provides a reference for further research on the functions of the HD-Zip family genes in physic nut and other species, and also provides important candidate genes for the genetic breeding of physic nut.

## Data availability statement

The original contributions presented in the study are included in the article/**Supplementary Material**. Further inquiries can be directed to the corresponding author.

## Author contributions

YHT conceived and designed the experiments; JP, JL, MZ, YT, SC, and YS performed experiments; QW and MZ analyzed the data; XB provided research advice; YHT wrote and revised the manuscript. All authors contributed to the article and approved the submitted version.
